# Supplementary figures and images for: Effectiveness of time-varying echo information for target geometry identification in bat-inspired human echolocation
Source: PLoS One. 2021 May 5;16(5):e0250517. doi: 10.1371/journal.pone.0250517 (PMC8099053; doi:10.1371/journal.pone.0250517)

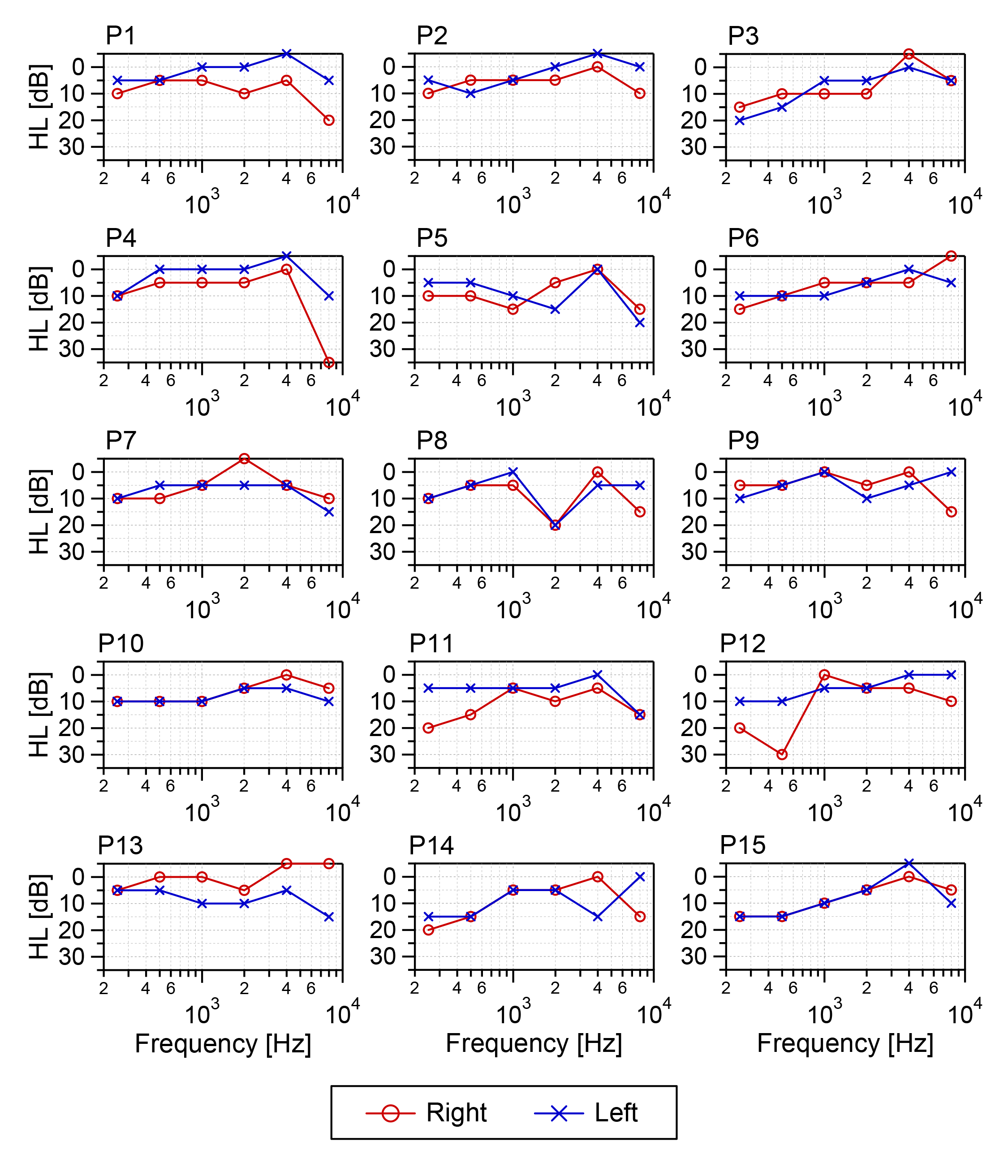

Supplement: S1 Fig — Red circles and blue cross marks indicate the hearing levels of the right and left ears of the 15 participants. (TIF) [file pone.0250517.s001.tif]

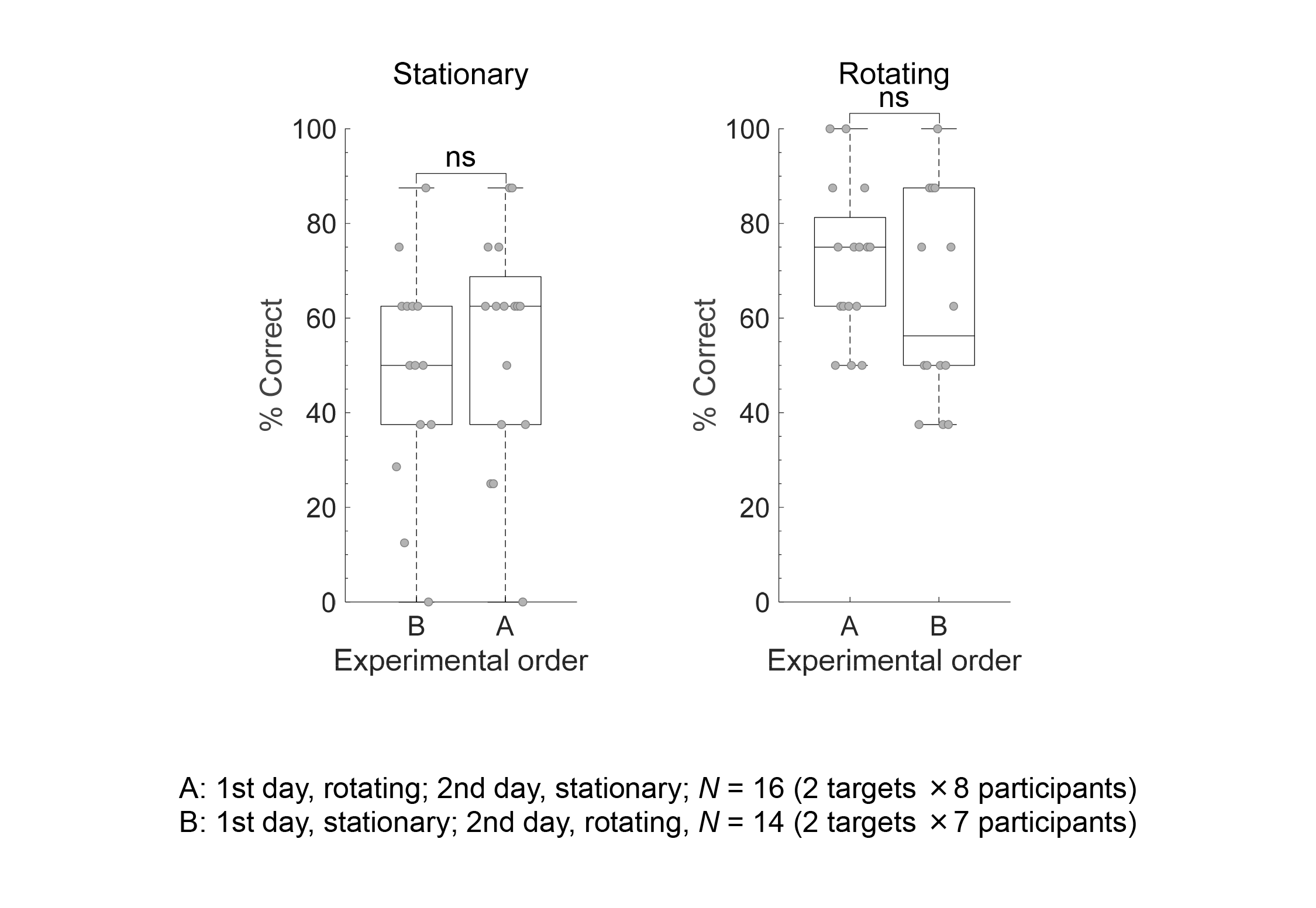

Supplement: S2 Fig — This figure shows box-and-whisker plots of the percentages of correct answers of the participants who participated in the experiment in experimental orders A (P1–P8) and B (P9–P15) for targets 1 and 2 in the test trials under the stationary (left) and rotating (right) conditions. The horizontal lines in the boxes indicate the median. The bottom and top of the boxes indicate the 25th (q1) and 75th (q3) percentiles, respectively. The bottom and top whiskers extend to the minimum and maximum data points within the range of [q1 − 1.5 × (q3 − q1)] to [q3 + 1.5 × (q3 − q1)]. The result of the logistic regression based on the GLMM is indicated by “ns” (non-significant). (TIF) [file pone.0250517.s002.tif]

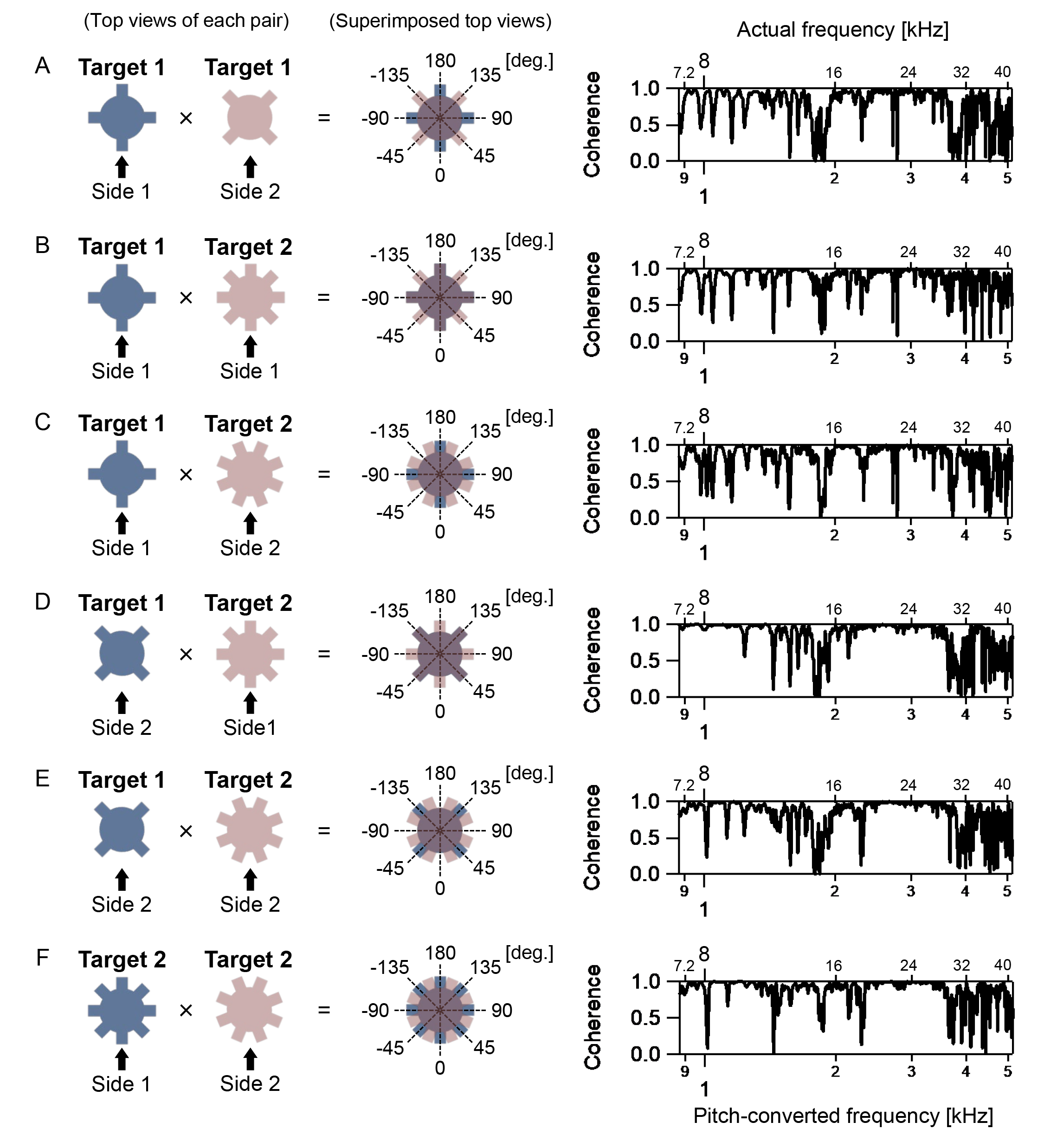

Supplement: S3 Fig — (A–F) denote the magnitude-squared coherence corresponding to the acoustic similarities among the pitch-converted binaural sounds to which P12 listened in the training trials under the stationary condition. The pitch-converted binaural sounds include the pitch-converted binaural echoes from sides 1 and 2 of targets 1 and 2. The magnitude-squared coherence was calculated for all six pairs: side 1 of target 1 and side 2 of target 1 (A), side 1 of target 1 and side 1 of target 2 (B), side 1 of target 1 and side 2 of target 2 (C), side 2 of target 1 and side 1 of target 2 (D), side 2 of target 1 and side 2 of target 2 (E), and side 1 of target 2 and side 2 of target 2 (F). The magnitude-squared coherence was calculated using fast Fourier transform (FFT) after averaging 324 readings of the pitch-converted binaural sounds (using the second to the last pitch-converted binaural sounds). The sampling frequency was 12 kHz, and the FFT window length was 4,096 points. The top horizontal logarithmic axes depict the actual frequency before pitch conversion. The middle panels display the superimposed top views of the two targets (light blue and pink targets), and the purple areas indicate the common shapes of the targets. The magnitude-squared coherence suggests that the similarities in timbre and/or pitch among the pitch-converted binaural echoes are not determined only by the type of target (1 or 2) and the shape of the side facing the sound source (convex or concave). (TIF) [file pone.0250517.s003.tif]
