# Supplementary material for: Effectiveness of time-varying echo information for target geometry identification in bat-inspired human echolocation
Source: PLoS One. 2021 May 5;16(5):e0250517. doi: 10.1371/journal.pone.0250517 (PMC8099053; doi:10.1371/journal.pone.0250517)
Supplement: Attachment — Submitted filename: plos review.docx [file pone.0250517.s007.docx]

Overall this is a nice manuscript with interesting findings. The results are well suited to address the question “What is it like to be a bat?” and are a potentially exciting addition to the field of auditory perception and discrimination. A revision should include more in-depth analysis of the participant variability in results and a careful interpretation of whether stationary echoes can be discriminated. This does not affect the main findings that time-varying acoustic features improve texture discrimination and target identification in human listeners. Other suggested revisions are mostly minor and related to structure and clarity of the methods section.

Major 1: The data in table 1 show a large amount of participant variability. This is not mentioned in the results and only slightly interpreted in the discussion. Figures to help visualize this would be very helpful. Perhaps an additional figure to show accuracy trajectory between training and test trials for each participant or revising Figure 4 to more easily see the distribution of accuracy data. With a sample size of fifteen, it would help with interpretation of the results to easily see if there are exceptional discriminators and non-discriminators in the data set. Additionally, there are several participants who perform (significantly?) below 50% accuracy in the stationary test. This should be carefully interpreted, as it indicates that the participants can discriminate something between the echoes produced by the targets, even if they are unable to correctly assign target identity. Perhaps with a longer training period they would be able to. They may drag the mean accuracy down to 50%, despite several participants also performing above chance level. This is mentioned in the discussion, but I think deserves a little more investigation. If they can successfully discriminate sounds, even without successfully assigning identity it would suggest that multiple time-varying views are not necessary, just helpful, for texture discrimination. Texture discrimination (Smoother vs. rougher, or even “is this different?”) is a related but slightly different task than target identification (1 vs. 2), even though they are presented as equivalent in the language of the discussion.

Major 2: It is unclear from the text why was the number of training trials set at 16. Frequently in animal models we set a criterion performance level rather than a fixed number of training trials because there is a large spread in learning times. Perhaps some participants would perform better with more training? It is also unclear why there were only 16 test trials. Was this the result of a power analysis? With a sample size of 15 participants, more test trials would help establish that the resulting mean was an accurate value, especially with a small effect size and high participant variability. I don’t doubt that the results are significant, but this choice needs to be clarified.

Minor: The methods felt a bit disjointed and would benefit from restructuring. While reading, I had several questions about the stimulus properties that were later answered in other sections of the methods. I would suggest describing in detail the properties of both the initial pulse and final playback signal earlier, either before or immediately after describing the equipment set up. It was hard to determine if the design was appropriate and the participant hearing range acceptable without knowing the signal characteristics. Initial pulse frequency range and sweep length, final pitched down pulse frequency range and sweep length, minimum and maximum decibel level of pulse and playback, IPI, and pulse number all should be clear and in one section.

Below are line by line questions I had while reading

Line 126: what is dB level of pulse emission? What was the dB level of playback? Was it consistent across participants or were participants able to set it? The relative amplitudes across frequencies for each target can be gathered from Figure 5, but a numeric value for min, max, and average for each target would be nice to see.

Line 138: I’m unclear on what “became the integral number means”

Line 220: were participants allowed to sample by playing the pulse sequence multiple times or only once?

Line 234: It would be helpful to put the speed of rotation in context of interpulse interval, i.e. x degrees between each pulse

Line 292-297: this is worded a bit confusingly. Do you mean that if the participants waited too long to start the pulse sequence the data were excluded? Was there a time limit on each trial?

Line 319: I think this sentence is missing a word

Line 415-422: were participants instructed in how to define time-varying vs not? It is not clear from the text whether the survey was descriptive or a checklist

Line 433: This needs more interpretation

Line 509: much of this presentation of by participant variability should be in the results
