# Supplementary material for: Effectiveness of time-varying echo information for target geometry identification in bat-inspired human echolocation
Source: PLoS One. 2021 May 5;16(5):e0250517. doi: 10.1371/journal.pone.0250517 (PMC8099053; doi:10.1371/journal.pone.0250517)
Supplement: Attachment — Submitted filename: final_plosone2021_2nd_response to reviewers.docx [file pone.0250517.s008.docx]

**Editor’s remarks to the author**

Thank you for submitting your manuscript to PLOS ONE. After careful consideration, we feel that it has merit but does not fully meet PLOS ONE’s publication criteria as it currently stands. Therefore, we invite you to submit a revised version of the manuscript that addresses the points raised during the review process.

This is an interesting study that explores human capability in extracting informative cues from echoes. As noted by the reviewers in details, the authors are encouraged to revise the manuscript and particularly address the issue of subject variability. The graphical displays chosen in the study do not adequately address this issue nor does the paper address the differences between participants and train/test with careful analysis. Furthermore, the authors are advised to revise the write-up as it can be better streamlined for readability and language editing. It contains number of superfluous sections and extremely long and verbose segments.

**Reply to the editor**

Thank you for your support. We revised the manuscript carefully to address the concerns of the reviewers. We hope that the revised manuscript will be appropriate for publication in PLOS ONE.

**Review Comments to the Author**

**Reviewer #1:**

**Comment 1:**

The summary data and variance are all available in the supplemental figures, but there is no visualization of raw data points used to arrive at the means. This is easily fixed either by the addition of a figure or a link to the raw data on a hosting service. If the data are unaccessible due to participant anonymity concerns, this should be stated.

**Acknowledgment:**

Thank you for the thorough review of the manuscript and for providing valuable comments and suggestions for improvement. We revised the manuscript based on the comments and suggestions.

**Revised point:**

Thank you for the comment. We visualized the performance of the participants and the results of the GLMM evaluations using boxplots (Fig 5). In addition, all data points were added to the boxplots.

**Reviewer #2:**

The authors explore the use of pitch converted echoes from echolocation signals to investigate the acoustic cues that may help identify object identity. The authors describe the use of time-varying information for correct object identification. The experiment is well thought of and interesting. I believe this work is of importance as it may prove to inspire the development of gadgets that may be used by visually impaired individuals for navigation.

**Acknowledgment:**

Thank you for the encouraging feedback and for examining the manuscript and providing valuable comments and suggestions for improvement. We have revised the manuscript based on the comments and suggestions.

**Comment 1:**

I have one major comment regarding the plotting of the data, bar graphs are not the most appropriate way to show this data as the variability between participants fades out this way (a variability which is evidenced in table one by some participants having very high percentages of correct answers, e.g. P1 and P12). This data should be plotted in box plots with all data points visible. I strongly suggest using box plots with all data points visible.

**Revised point:**

Thank you for the suggestion. We illustrated the performance of the participants using boxplots (Fig 5). All data points were added in the boxplots.

**Comment 2:**

Additionally, I have a few comments that would improve the clarity of the manuscript:

Line 2 - What do the authors mean by appropriately designed ultrasounds? Nothing has been designed in evolution.... Re-word.

**Revised point:**

Thank you for the comment. The expression was used to explain the two following points: bats (1) adapted echolocation signals (ultrasounds) to locate habitat environments and as a foraging strategy, and (2) changed the acoustic features of echolocation signals to adapt to situations and purposes. However, as indicated, readers may find the expression difficult to understand. Therefore, we revised the statement as follows:

**Page 2, Line 2:**

**Bats use echolocation through flexible active sensing via ultrasounds to recognize environments suitable for their habitat and foraging.**

**Comment 3:**

Line 8 (and others) - What do the authors mean by “an echolocation signal, including ultrasound”? Anything above 20kHZ is ultrasound. I recommend just referring to “synthetic echolocation signal” and defining it as it is done in the “echolocation signal” part of the methods.

**Revised point:**

Thank you for the suggestion. We reworded the expression throughout the manuscript accordingly. In addition, the Abstract and Introduction sections cite the highest frequency of the synthetic echolocation signal to highlight the necessity of pitch conversion.

**Page 2, Line 8:**

**Broadband frequency-modulated pulses with a short inter-pulse interval (16 ms) were used as a synthetic echolocation signal. Such pulses mimic buzz signals emitted by bats for echolocation prior to capturing prey.**

**Page 5, Line 92:**

**Because the signal included high-frequency signals up to 41 kHz, the signal and echoes from the target were recorded using the MDH, which was also used in the previous study (17).**

For the same reason, we revised the description of our previous study as follows:

**Page 4, Line 50:**

**The pitch of binaural echoes was down-converted after a miniature dummy head (MDH) (18) captured echoes because the signals included high-frequency signals up to 35 kHz.**

We also revised the title because the study does not require emphasis of the word “ultrasonic”.

**Title**

**Effectivity of time-varying echo information acquired by bat-inspired acoustic sensing in identifying target geometry using human echolocation**

**Comment 4:**

Line 34 - Again, not designed. Re-word.

**Revised point:**

Thank you for the suggestion. We reworded the expression as follows.

**Page 3, Line 34:**

**Such flexible acoustic sensing using echolocation signals makes up advanced sonar behaviors in animals (10, 11).**

**Comment 5:**

Line 71 - “acquired multiple sensing”, this sentence needs re-wording, it is unclear.

**Revised point:**

Thank you for the suggestion. We revised the sentence as follows.

**Page 5, Line 77:**

**In summary, previous studies suggested that FM bats use “time-varying echo information” for object recognition and texture discrimination by emitting echolocation signals repeatedly toward objects from various angles and positions and listening to time-varying echoes.**

**Comment 6:**

Line 164 - replace “were not up to” with “were not above”

**Revised point:**

Thank you for the suggestion. We revised the expression as follows.

**Page 9, Line 184:**

**Nevertheless, the data for P4 were included in the analysis because the pitch-converted binaural sounds presented in the experiment were not above 8 kHz (approximately 1–5 kHz).**

**Comment 7:**

Line 152 - One graph with the audiograms should be included as supplementary figures.

**Revised point:**

Thank you for the suggestion. We included the audiograms as supporting information (S1 Fig) and slightly revised the Participants section as follows.

**Page 9, Line 182:**

**The pure tone thresholds in the left and right ears of the participants were within a hearing level of 30 dB from 250 Hz to 8,000 Hz, except for P4, who exhibited a pure tone threshold of 35 dB at 8,000 Hz in the right ear (S1 Fig).**

**Comment 8:**

Line 251 - The section echolocation signal should be brought up to around line 150. In line 123 they only specify an upper limit of 48kHz (line 123). Also at that point it is unclear if it has harmonics of if it is a single FM sweep. These are important parameters that need to be addressed earlier. Bringing the section up would avoid this problem.

**Revised point:**

Thank you for the suggestion. We moved “Synthetic echolocation signal” as a subsection under “Sensing system” section. We omitted the description of the upper limit to prevent confusion.

**Comment 9:**

Line 256 to 261 - Unclear sentence. How fast does the object rotate? From this sentence I understand that the target rotates at ~0.27 rpm, thus one rotation takes approximately 164 s. If this is correct, this should be stated clearly before this sentence.

**Revised point:**

Thank you for the suggestion. The target rotated at 2.95 rpm. We added the information under the “Experimental conditions” section.

**Page 12, Line 245:**

**Under the rotating condition, the target rotated horizontally with the electric turntable at a rate of approximately 2.95 rpm (0.28° between pulses) in a counterclockwise direction (Fig 4, red arrow).**

**Comment 10:**

Line 270 - When was the feedback presented? Simultaneous to the pitch converted echo? Before? Or after? This is VERY important information in particular for the results in figures S7 and S8.

**Revised point:**

Thank you for pointing out this aspect. The feedback was presented to the participants immediately after their responses. We added the information in the “Procedure” section as follows.

**Page 13, Line 269:**

**The answer feedback from the training trials was presented as 3D graphics of the targets using the experiment control software on the display of the notebook computer, which was presented to the participants immediately after their responses.**

**Comment 11:**

Line 274 to 276 - Unclear. Place the target where?

**Revised point:**

Thank you for this observation. The targets were placed on the stationary or rotating turntable in the experiment. We revised the sentence as follows.

**Page 13, Line 274:**

**The experimenter stayed in chamber 2 to remotely monitor the display of the notebook computer in chamber 1 and to place the targets on the stationary or rotating turntable in chamber 2 accordingly.**

**Comment 12:**

Line 292 - “In case the pulses…” re words to “In the case that the pulses…”

**Revised point:**

Thank you for the suggestion. We revised the sentence as follows.

**Page 14, Line 290:**

**In the case that the pulses and echoes were recorded at less than 4/5 of the sensing duration due to a delay in tapping device A for pulse emission, the response was excluded from the experimental data to ensure data reliability.**

**Comment 13:**

Line 368 - Also, figures S4, S5 and S6 should be sub-panels of figure 4 and should also show all data points. It is not only important if there is a difference between stationary and rotating, but one could argue that it is more important to evaluate if the answers are significantly different than chance. Same for figure S7 and S8.

**Revised point:**

Thank you for the suggestion. We added the previous supporting figures (S3–S8 Figs), which demonstrated the results of the GLMM evaluations in Fig 5B as sub-panels for Fig 5A.

**Comment 14:**

Line 442 - Authors should specify in the ESD is calculated based on an average of several echoes, as background noise may amplify notches in a single echo and thus accentuate differences.

**Revised point:**

Thank you for the suggestion. We recalculated the ESDs and background noise floor on the basis of averaged sounds and revised the figure (Fig 7) and caption as follows.

**Page 22, Line 471:**

**Each ESD was calculated by FFT after averaging the pitch-converted binaural sounds by 324 times (using the second to the last pitch-converted binaural sounds). The background noise floor was also calculated by FFT after averaging the sounds by 324 times.**

**Comment 15:**

Line 459 - How are the authors differentiating between “correct answer” and mental imagery? This is unclear. If the participants report hearing a specific target rotated how is this different from reporting they imagine a rotating target. Furthermore, since the participants were shown the target before-hand, the imagery does not add any information. This would be much more interesting if the participants were asked to describe what they imagine without having previously seen the targets or told they could either rotate or be stationary. This section of the results doesn’t add much and is confusing. Could be cut.

**Revised point:**

Thank you for this observation. We omitted the descriptions and will design appropriate experiments to examine mental imagery in future studies.

**Comment 16:**

Line 480 - The authors do not speculate as to why in test conditions without feedback the participants had higher accuracy in the discrimination than in the training conditions. During training they are above chance for the rotating trials but not the stationary trials, yet these two are not significantly different from each other…. Is it because the feedback is present constantly? This should be tackled in the discussion.

**Revised point:**

Thank you for the suggestion. We added the following discussion.

**Page 24, Line 506:**

**The study determined the number of training trials (16 trials) based on a restriction of an experimental duration (up to 2 h). However, it could be increased by conducting the experiment in each condition across several days. Training and test performance may be improved by increasing the frequency of training. However, despite more training, the possibility exists that several participants will exhibit extremely low performance in the test trials under the stationary condition. For instance, P15 under the stationary condition displayed an extremely low performance in the test trials (18.8%) but not in the training trials (68.8%) (Fig 5A). By constantly checking the answer feedback, the participants may familiarize the associations between the non-time-varying echo information and target geometries under the stationary condition. In contrast, although the test performance of P13 and P14 under the rotating condition was less than the chance level, no participants exhibited extremely low performance in the test trials (Fig 5A, right panel). These results suggest that time-varying echo information is easier for humans to associate with target geometries and for familiarizing the associations without answer feedback.**

**Comment 17:**

Line 506 - Again, it is not clear if the feedback was present all the time during echo presentation or only before or afterwards.

**Revised point:**

Thank you for pointing out this aspect. We added a description for answer feedback based on Comment 10.

**Reviewer #3:**

The research reported in this manuscript addresses an interesting question: Does the rotation of an ensonified target aid listeners in making echo-based classifications. However, there are serious issues with the results and the presentation of this work that should be addressed in order to make the manuscript suitable for publication:

**Acknowledgment:**

Thank you for the thorough review of the manuscript and for providing valuable comments and suggestions for improvement. We revised the manuscript accordingly.

**Comment 1:**

1. The results show a big difference between training and test trials. What is presented as the main finding of the research, i.e., subjects can make the discrimination only with rotation, exists only in the test results, but not in the training results. In the transition from training (with feedback) to testing (w/o feedback), the subjects somehow got a lot worse at the stationary targets and quite a bit better for the rotating targets. Hence, the main difference here seems to be due to an interaction between experimental condition (feedback vs. no-feedback, naive vs. experienced) and target condition (stationary vs. rotating) and not due to target condition alone. The results should be presented by stating this clearly throughout the manuscript, i.e., in abstract, results, and discussion.

**Revised point:**

Thank you. We revised the Abstract, Results, and Discussion sections as per the suggestion.

We described the performance of the participants in the training and test trials separately in the Results section as follows.

**Page 17, Line 360:**

**Fig 5A and B present the target geometry identification performance of the participants and the results of the GLMM evaluation, respectively. Although the majority of participants (12/15) in the training trials displayed performance above the chance level under the stationary and rotating conditions (Fig 5A, left panel), participants exhibited extremely high performance only in the rotating condition (P1: 81.3%; P12: 87.5%). The GLMM evaluations indicated that the participants in the training trials were more likely to identify the target geometries correctly than chance performance in the rotating condition (Fig 5B, middle left panel; β = 0.533 ± 0.186, z = 2.863, p < 0.01) but not in the stationary condition (Fig 5B, top left panel; β = 0.360 ± 0.185, z = 1.946, p = 0.052). Furthermore, the GLMM evaluation indicated that the participants in the training trials were not more likely to identify the target geometries correctly in the rotating condition than in the stationary condition (Fig 5B, bottom left panel; β = 0.174 ± 0.189, z = 0.921, p = 0.357).**

**Page 18, Line 392:**

**Although the target geometry identification performance of the participants in the test trials displayed more variations that those for the training trials under the stationary and rotating conditions (Fig 5A, right panel), the median of the proportions of correct responses increased only in the rotating condition [stationary: 56.3% (training), 56.3% (test); rotating: 62.5% (training), 68.8% (test)]. Under the stationary condition, the number of participants with performance less than or equal to the chance level increased in the test trials (training: 3/15 participants; test: 6/15 participants), and the test performance of P2 (25.0%), P8 (18.8%), P10 (37.5%), and P15 (18.8%) was extremely low. Under the rotating condition, the number of participants with extremely high performance (above 80%) increased in the test trials (training: 2/15 participants; test: 4/15 participants), and P1 and P12 displayed the highest test performance (93.8%), who also showed extremely high performance in the training trials. The GLMM evaluations indicated that the participants in the test trials were more likely to identify the geometries correctly than chance performance in the rotating condition (Fig 5B, middle right panel; β = 0.750 ± 0.189, z = 3.964, p < 0.001) but not in the stationary condition (Fig 5B, top right panel; β = 0.076 ± 0.183, z = 0.413, p = 0.680). Moreover, the GLMM evaluation indicated that the participants in the test trials were more likely to identify the geometries correctly in the rotating condition than in the stationary condition (Fig 5B, bottom right panel; β = 0.700 ± 0.193, z = 3.619, p < 0.001).**

In the first paragraph of the Discussion section, we discussed the differences in the performance between the stationary and rotating conditions and between the training and test trials as follows.

**Page 23, Line 482:**

**In the training and test trials, the participants identified the two target geometries in the presence of time-varying echo information as the acoustic cue (rotating condition) but failed to do so in the absence of time-varying echo information (stationary condition) (Fig 5B, top and middle panels). Given the result of the hearing survey (Table 1), the results suggest that sighted people can identify target geometries using time-varying patterns according to echo intensity, timbre, and/or pitch by sensing targets from different angles using buzz-like FM signals. However, performance significantly differed between the stationary and rotating conditions only in the test trials (Fig 5B, bottom panels). This finding suggests that time-varying echo information is effective for target geometry identification by human echolocation especially when echolocators cannot obtain answer feedback during sensing.**

We also revised the Abstract on the basis of the revised Results and Discussion sections as follows.

**Page 2, Line 16:**

**In both training and test trials with (training) and without (test) answer feedback immediately after the participants answered, the participants identified the geometries under the rotating condition. The majority reported the use of time-varying patterns in echo intensity, timbre, and/or pitch under the rotating condition. The results suggested that using time-varying patterns in echo intensity, timbre, and/or pitch enables humans to identify target geometries. However, performance significantly differed between the stationary and rotating conditions only in the test trials. This difference suggested that time-varying echo information is effective for identifying target geometry through human echolocation especially when echolocators are unable to obtain answer feedback during sensing.**

**Comment 2:**

2. The manuscript presents the task as "texture discrimination". "Texture" is a surface property, e.g., smooth or rough, that refers to geometrical detail on a scale that is much smaller than that of the overall geometry of the object. The distinguishing feature of the two targets used here (number of "gear teeth") is on the same scale as the target itself. The "gear teeth" are also not small compared to the wavelengths used - at least for the upper portion of the frequency band. Hence, this task should be cast as a "target geometry" discrimination.

**Revised point:**

Thank you for the suggestion. With respect to “texture” used to describe the current experiment, we decided to use “target geometry”.

**Comment 3:**

3. The manuscript is unnecessarily verbose, e.g., large portions of the discussion are repetitions of the results. In addition, the language is rife with clumsy wordings ("sonar behaviors in biosonar animals"). It would be best to have this looked over by a native speaker.

**Revised point:**

We apologize for the verbose and inappropriate English expressions. We sought the help of a native English speaker with proofreading and hope that significant improvements were made.

**Attached review comments**

Overall this is a nice manuscript with interesting findings. The results are well suited to address the question “What is it like to be a bat?” and are a potentially exciting addition to the field of auditory perception and discrimination. A revision should include more in-depth analysis of the participant variability in results and a careful interpretation of whether stationary echoes can be discriminated. This does not affect the main findings that time-varying acoustic features improve texture discrimination and target identification in human listeners. Other suggested revisions are mostly minor and related to structure and clarity of the methods section.

**Acknowledgment:**

Thank you for the thorough review of the manuscript and for providing valuable comments and suggestions for improvement. We revised the manuscript accordingly.

**Comment 1:**

Major 1: The data in table 1 show a large amount of participant variability. This is not mentioned in the results and only slightly interpreted in the discussion. Figures to help visualize this would be very helpful. Perhaps an additional figure to show accuracy trajectory between training and test trials for each participant or revising Figure 4 to more easily see the distribution of accuracy data. With a sample size of fifteen, it would help with interpretation of the results to easily see if there are exceptional discriminators and non-discriminators in the data set. Additionally, there are several participants who perform (significantly?) below 50% accuracy in the stationary test. This should be carefully interpreted, as it indicates that the participants can discriminate something between the echoes produced by the targets, even if they are unable to correctly assign target identity. Perhaps with a longer training period they would be able to. They may drag the mean accuracy down to 50%, despite several participants also performing above chance level. This is mentioned in the discussion, but I think deserves a little more investigation. If they can successfully discriminate sounds, even without successfully assigning identity it would suggest that multiple time-varying views are not necessary, just helpful, for texture discrimination. Texture discrimination (Smoother vs. rougher, or even “is this different?”) is a related but slightly different task than target identification (1 vs. 2), even though they are presented as equivalent in the language of the discussion.

**Revised point:**

Thank you for the insightful comments.

We illustrated the performance of the participants using boxplots in Fig 5. Several participants displayed extremely high and low levels of performance under the rotating and stationary conditions. Therefore, we described their performance in the Results section. Specifically, we explained that time-varying versus non-time-varying echo information is more robust and reasonable as an acoustic cue for identifying target geometry for the following reasons: (1) association between echoes and target geometries, (2) familiarization of associations without answer feedback, and (3) duration of training. These revisions are reflected in the following text.

**Page 17, Line 361:**

**Although the majority of participants (12/15) in the training trials displayed performance above the chance level under the stationary and rotating conditions (Fig 5A, left panel), participants exhibited extremely high performance only in the rotating condition (P1: 81.3%; P12: 87.5%).**

**Page 18, Line 396:**

**Under the stationary condition, the number of participants with performance less than or equal to the chance level increased in the test trials (training: 3/15 participants; test: 6/15 participants), and the test performance of P2 (25.0%), P8 (18.8%), P10 (37.5%), and P15 (18.8%) was extremely low. Under the rotating condition, the number of participants with extremely high performance (above 80%) increased in the test trials (training: 2/15 participants; test: 4/15 participants), and P1 and P12 displayed the highest test performance (93.8%), who also showed extremely high performance in the training trials.**

**Page 23, Line 493:**

**In the stationary condition, although P2, P8, P10, and P15 exhibited extremely low performance (less than 40%) in the test trials (Fig 5A, right panel), P2 and P15 exhibited training performance above the chance level (Fig 5A, left panel). Thus, P2 and P15 under the stationary condition may be able to discriminate differences among the echoes but unable to associate the echoes with the target geometries correctly. The type of the target (target 1 or 2) and the shape of the side facing the sound source (convex or concave) do not solely determine the similarities in timbre and/or pitch among the pitch-converted binaural echoes (Fig 7 and S3 Fig). This aspect may be the reason for the difficulty of the participants in associating non-time-varying echo information with target geometries, even between the two targets, under the stationary condition. One can easily predict that performance would decline using more target geometries compared with those in the current experiment. These results suggest that non-time-varying echo information is unsuitable for target geometry identification.**

**The study determined the number of training trials (16 trials) based on a restriction of an experimental duration (up to 2 h). However, it could be increased by conducting the experiment in each condition across several days. Training and test performance may be improved by increasing the frequency of training. However, despite more training, the possibility exists that several participants will exhibit extremely low performance in the test trials under the stationary condition. For instance, P15 under the stationary condition displayed an extremely low performance in the test trials (18.8%) but not in the training trials (68.8%) (Fig 5A). By constantly checking the answer feedback, the participants may familiarize the associations between the non-time-varying echo information and target geometries under the stationary condition. In contrast, although the test performance of P13 and P14 under the rotating condition was less than the chance level, no participants exhibited extremely low performance in the test trials (Fig 5A, right panel). These results suggest that time-varying echo information is easier for humans to associate with target geometries and for familiarizing the associations without answer feedback.**

**P4 displayed extremely high performance (81.3%) in the test trials under the rotating condition (Fig 5A, right panel). However, the training performance of P4 was the same as the chance level (Fig 5A, left panel). Thus, P4 may require more time to grasp the technique to associate the time-varying echo information and target geometries compared with P1, P11, and P12, who also exhibited extremely high performance in the test trials (Fig 5A). This finding implies that a substantial amount of training may not always be necessary for target geometry identification using time-varying echo information. However, once the technique of associating time-varying echo information with target geometries is grasped, it may become robust. This notion may be involved with the use of time-varying information for sensing through other sensory modalities (e.g., haptic sense) in daily life.**

We also added descriptions in the Introduction to emphasize the difference between discrimination and identification.

**Page 4, Line 56:**

**In the previous study, participants without vision loss discriminated texture by listening to the timbre of echoes, which suggested that texture can be discriminated using synthetic echolocation signals with suitable frequency bands and time–frequency structures (17). However, the previous experiment on texture discrimination was performed using a three-interval, two-alternative forced-choice task, which implied that the participants discriminated texture by simply determining whether the first and second (or second and third) sound stimuli were different. Therefore, whether humans can also “identify” texture only by designing synthetic echolocation signals remains unclear.**

**Comment 2:**

Major 2: It is unclear from the text why was the number of training trials set at 16. Frequently in animal models we set a criterion performance level rather than a fixed number of training trials because there is a large spread in learning times. Perhaps some participants would perform better with more training? It is also unclear why there were only 16 test trials. Was this the result of a power analysis? With a sample size of 15 participants, more test trials would help establish that the resulting mean was an accurate value, especially with a small effect size and high participant variability. I don’t doubt that the results are significant, but this choice needs to be clarified.

**Revised point:**

Thank you for the comment. The number of training trials (16 trials) was determined on the basis of the restriction of experimental duration (up to 2 h). We added the rational for this choice in the Discussion section (please see text below). However, we were unable to conduct the experiment under each condition for several days. In future studies, we will determine carefully the number of test trials according to the number of participants and data variability as per your advice.

**Page 24, Line 506:**

**The study determined the number of training trials (16 trials) based on a restriction of an experimental duration (up to 2 h). However, it could be increased by conducting the experiment in each condition across several days. Training and test performance may be improved by increasing the frequency of training.**

**Comment 3:**

Minor: The methods felt a bit disjointed and would benefit from restructuring. While reading, I had several questions about the stimulus properties that were later answered in other sections of the methods. I would suggest describing in detail the properties of both the initial pulse and final playback signal earlier, either before or immediately after describing the equipment set up. It was hard to determine if the design was appropriate and the participant hearing range acceptable without knowing the signal characteristics. Initial pulse frequency range and sweep length, final pitched down pulse frequency range and sweep length, minimum and maximum decibel level of pulse and playback, IPI, and pulse number all should be clear and in one section.

**Revised point:**

Thank you for the suggestion. We moved “Synthetic echolocation signal” under the “Sensing system” section to enhance the flow of idea. We also added the sound pressure levels of the signal emission and playback under the “Sensing system” section as follows.

**Page 8, Line 149:**

**The sound pressure level (Lp) of the signal was 117 dB and was situated 10 cm away from the loudspeaker.**

**Page 8, Line 161:**

**The pitch-converted binaural sounds were presented to the participants through headphones (MDR-CD900ST; Sony Corporation, Tokyo, Japan) with sound pressure levels (Lp) ranging from 56 dB to 69 dB at the entrance of the right ear canal.**

**Comment 4:**

Below are line by line questions I had while reading

Line 126: what is dB level of pulse emission? What was the dB level of playback? Was it consistent across participants or were participants able to set it? The relative amplitudes across frequencies for each target can be gathered from Figure 5, but a numeric value for min, max, and average for each target would be nice to see.

**Revised point:**

Thank you for the comment. We added the sound pressure level of the synthetic echolocation signal under the “Sensing system” section. The participants were instructed to refrain from changing the volume of the pitch-converted binaural sounds. In addition, we included this information in the “Experimental system and general task” section. The sound pressure levels of the pitch-converted binaural sounds were also included in the “Acoustic cues for target geometry identification” section. Please find below the following revisions.

**Page 8, Line 149:**

**The sound pressure level (Lp) of the signal was 117 dB and was situated 10 cm away from the loudspeaker.**

**Page 11, Line 233:**

**The participants were instructed to refrain from adjusting the volume of the pitch-converted binaural sounds but were confirmed that the sounds were within acceptable range.**

**Page 19, Line 420:**

**Although the sound pressure levels (Lp) of the pitch-converted binaural sounds (at the entrance of the right ear canal) in target 1 (maximum: 67.2 ± 0.06 dB [mean ± standard error]; minimum: 58.5 ± 0.04 dB; mean: 63.1 ± 0.04 dB; N = 120 [8 trials × 15 participants]) were higher than those in target 2 (maximum: 65.9 ± 0.04 dB; minimum: 57.9 ± 0.06 dB; mean: 61.9 ± 0.04 dB; N = 120 [8 trials × 15 participants]), the difference was small (less than 1.3 dB).**

**Comment 5:**

Line 138: I’m unclear on what “became the integral number means”

**Revised point:**

We apologize for the confusion. The pitch conversion rate was 1/8 instead of 1/7 because we opted to round the number from 13,714 to 12,000 for the sample rate to improve the accuracy of the playback as much as possible. We revised the expression as follows.

**Page 8, Line 160:**

**The pitch conversion rate used was 1/8 instead of 1/7 to round off the number from 13,714 to 12,000 for the sample rate.**

**Comment 6:**

Line 220: were participants allowed to sample by playing the pulse sequence multiple times or only once?

**Revised point:**

The participants were allowed to tap the Android devices once for each sensing. We revised the sentence as follows.

**Page 11, Line 229:**

**The participants initiated sensing by tapping the Android devices once after the notebook computer (Inspiron 7380; Dell Inc., TX, USA) placed in chamber 1 displayed the instruction to begin sensing.**

**Comment 7:**

Line 234: It would be helpful to put the speed of rotation in context of interpulse interval, i.e. x degrees between each pulse

**Revised point:**

Thank you for the suggestion. We added the following statement.

**Page 12, Line 245:**

**Under the rotating condition, the target rotated horizontally with the electric turntable at a rate of approximately 2.95 rpm (0.28° between pulses) in a counterclockwise direction (Fig 4, red arrow).**

**Comment 8:**

Line 292-297: this is worded a bit confusingly. Do you mean that if the participants waited too long to start the pulse sequence the data were excluded? Was there a time limit on each trial?

**Revised point:**

Thank you for pointing out this aspect. The participants tapped Android device A per pulse emission (5.2 s) immediately after tapping Android device B because Android device B employs a limited time for 6.052 s per sensing during recording. We revised the description as follows.

**Page 11, Line 229:**

**The participants initiated sensing by tapping the Android devices once after the notebook computer (Inspiron 7380; Dell Inc., TX, USA) placed in chamber 1 displayed the instruction to begin sensing. The participants tapped device A for pulse emission (5.2 s) immediately after tapping device B to listen to all pitch-converted binaural sounds because device B employs a limit of 6.052 s per each sensing during recording.**

**Comment 9:**

Line 319: I think this sentence is missing a word

**Revised point:**

Thank you for the suggestion. We revised the sentence as follows.

**Page 15, Line 317:**

**Building on DeLong et al. (22), the previous study (17) conducted a hearing survey for sighted participants to examine the effective acoustic cues used to discriminate shape, texture, and material through synthetic echolocation signals.**

**Comment 10:**

Line 415-422: were participants instructed in how to define time-varying vs not? It is not clear from the text whether the survey was descriptive or a checklist

**Revised point:**

Thank you for the comment. The participants provided descriptions of acoustic cues in free form. We added the description as follows.

**Page 15, Line 321:**

**The participants were encouraged to describe the acoustic cues they used to identify the target geometries using their own words.**

**Comment 11:**

Line 433: This needs more interpretation

**Revised point:**

Thank you for pointing out this aspect. We revised the sentence and clarified the interpretation by adding the following example.

**Page 22, Line 452:**

**Cross marks indicate that the participants did not use the echo information. For example, P1 did not use time-varying echo information as an acoustic cue under the stationary condition.**

**Comment 12:**

Line 509: much of this presentation of by participant variability should be in the results

**Revised point:**

Thank you for the suggestion. We described participant variability in the Result section as follows.

**Page 18, Line 396:**

**Under the stationary condition, the number of participants with performance less than or equal to the chance level increased in the test trials (training: 3/15 participants; test: 6/15 participants), and the test performance of P2 (25.0%), P8 (18.8%), P10 (37.5%), and P15 (18.8%) was extremely low.**
