# Supplementary material for: Effectiveness of time-varying echo information for target geometry identification in bat-inspired human echolocation
Source: PLoS One. 2021 May 5;16(5):e0250517. doi: 10.1371/journal.pone.0250517 (PMC8099053; doi:10.1371/journal.pone.0250517)
Supplement: Attachment — Submitted filename: final_plos2021_3rd_response to reviewers.docx [file pone.0250517.s009.docx]

**Journal Requirements:**

Please review your reference list to ensure that it is complete and correct. If you have cited papers that have been retracted, please include the rationale for doing so in the manuscript text, or remove these references and replace them with relevant current references. Any changes to the reference list should be mentioned in the rebuttal letter that accompanies your revised manuscript. If you need to cite a retracted article, indicate the article’s retracted status in the References list and also include a citation and full reference for the retraction notice.

**Reply:**

Thank you for your comment. We reviewed and revised the reference list as follows. We also confirmed that the references were not retracted.

**Revised point:**

**1.	Neuweiler G. Foraging, echolocation and audition in bats. Naturwissenschaften. 1984;71:446-455.**

**2.	Schnitzler H-U, Kalko EKV. Echolocation by insect-eating bats. Bioscience. 2001;51(7):557-569.**

**3.	Surlykke A, Moss CF. Echolocation behavior of big brown bats, *Eptesicus fuscus*, in the field and the laboratory. J Acoust Soc Am. 2000;108(5):2419-2429.**

**4.	Hiryu S, Hagino T, Fujioka E, Riquimaroux H, Watanabe Y. Adaptive echolocation sounds of insectivorous bats, *Pipistrellus abramus*, during foraging flights in the field. J Acoust Soc Am. 2008;124(2):EL51-EL56.**

**5.	Hiryu S, Shiori Y, Hosokawa T, Riquimaroux H, Watanabe Y. On-board telemetry of emitted sounds from free-flying bats: compensation for velocity and distance stabilizes echo frequency and amplitude. J Comp Physiol A. 2008;194:841-851.**

**6.	Fujioka E, Mantani S, Hiryu S, Riquimaroux H, Watanabe Y. Echolocation and flight strategy of Japanese house bats during natural foraging, revealed by a microphone array system. J Acoust Soc Am. 2011;129(2):1081-1088.**

**7.	Jakobsen L, Surlykke A. Vespertilionid bats control the width of their biosonar sound beam dynamically during prey pursuit. Proc Natl Acad Sci. 2010;107(31):13930-13935.**

**8.	Fujioka E, Aihara I, Watanabe S, Sumiya M, Hiryu S, Simmons JA, et al. Rapid shifts of sonar attention by *Pipistrellus abramus* during natural hunting for multiple prey. J Acoust Soc Am. 2014;136(6):3389-3400.**

**9.	Simmons JA, Hiryu S, Shriram U. Biosonar interpulse intervals and pulse-echo ambiguity in four species of echolocating bats. J Exp Biol. 2019;222:jeb195446.**

**10.	Fujioka E, Aihara I, Sumiya M, Aihara K, Hiryu S. Echolocating bats use future-target information for optimal foraging. Proc Natl Acad Sci. 2016;113(17):4848-4852.**

**11.	Sumiya M, Fujioka E, Motoi K, Kondo M, Hiryu S. Coordinated control of acoustical field of view and flight in three-dimensional space for consecutive capture by echolocating bats during natural foraging. PLoS ONE. 2017;12(1):e0169995.**

**12.	Kolarik AJ, Cirstea S, Pardhan S, Moore BCJ. A summary of research investigating echolocation abilities of blind and sighted humans. Hear Res. 2014;310:60-68.**

**13.	Thaler L, Goodale MA. Echolocation in humans: an overview. WIREs Cogn Sci. 2016;7:382-393.**

**14.	Thaler L, De Vos H, Kish D, Antoniou M, Baker CJ, Hornikx MCJ. Human click-based echolocation of distance: superfine acuity and dynamic clicking behaviour. J Assoc Res Otolaryngol. 2019;20:499-510.**

**15.	Milne JL, Goodale MA, Thaler L. The role of head movements in the discrimination of 2-D shape by blind echolocation experts. Atten Percept Psychophys. 2014;76:1828-1837.**

**16.	Thaler L, Vos RD, Kish D, Antoniou M, Baker C, Hornikx M. Human echolocators adjust loudness and number of clicks for detection of reflectors at various azimuth angles. Proc R Soc B. 2018;285:20172735.**

**17.	Sumiya M, Ashihara K, Yoshino K, Gogami M, Nagatani Y, Kobayasi KI, et al. Bat-inspired signal design for target discrimination in human echolocation. J Acoust Soc Am. 2019;145(4):2221-2236.**

**18.	Uchibori S, Sarumaru Y, Ashihara K, Ohta T, Hiryu S. Experimental evaluation of binaural recording system using a miniature dummy head. Acoust Sci & Tech 2015;36(1):42-45.**

**19.	Von Helversen D, Von Helversen O. Object recognition by echolocation: a nectar-feeding bat exploiting the flowers of a rain forest vine. J Comp Physiol A. 2003;189:327-336.**

**20.	Falk B, Williams T, Aytekin M, Moss CF. Adaptive behavior for texture discrimination by the free-flying big brown bat, *Eptesicus fuscus*. J Comp Physiol A. 2011;197:491-503.**

**21.	Suzuki Y, Takeshima H. Equal-loudness-level contours for pure tones. J Acoust Soc Am. 2004;116(2):918-933.**

**22.	DeLong CM, Au WW, Stamper SA. Echo features used by human listeners to discriminate among objects that vary in material or wall thickness: Implications for echolocating dolphins. J Acoust Soc Am. 2007;121(1):605-617.**

**23.	Schall R. Estimation in generalized linear models with random effects. Biometrika. 1991;78(4):719-727.**

**25.	Schnitzler H-U, Moss CF, Denzinger A. From spatial orientation to food acquisition in echolocating bats. Trends Ecol Evol. 2003;18(8):386-394.**

**26.	Au WW, Martin DW. Insights into dolphin sonar discrimination capabilities from human listening experiments. J Acoust Soc Am. 1989;86(5):1662-1670.**

**27.	Yu C, Luo J, Wohlgemuth M, Moss CF. Echolocating bats inspect and discriminate landmark features to guide navigation. J Exp Biol. 2019;222:jeb191965.**

**Additional Editor Comments:**

The authors are commanded on their revision of the manuscript. As noted by reviewers, all major edits and comments were addressed. The authors are encouraged to do one more revision of the manuscript focusing on language editing to improve readability.

**Reply to the editor:**

Thank you for your support. Considering your suggestion, we revised the manuscript accordingly, focusing on the language to improve readability.

**Reviewer’s Responses to Questions:**

1. If the authors have adequately addressed your comments raised in a previous round of review and you feel that this manuscript is now acceptable for publication, you may indicate that here to bypass the “Comments to the Author” section, enter your conflict of interest statement in the “Confidential to Editor” section, and submit your "Accept" recommendation.

Reviewer #1: All comments have been addressed

Reviewer #2: All comments have been addressed

Reviewer #3: All comments have been addressed

2. Is the manuscript technically sound, and do the data support the conclusions?

The manuscript must describe a technically sound piece of scientific research with data that supports the conclusions. Experiments must have been conducted rigorously, with appropriate controls, replication, and sample sizes. The conclusions must be drawn appropriately based on the data presented.

Reviewer #1: Yes

Reviewer #2: Yes

Reviewer #3: Yes

3. Has the statistical analysis been performed appropriately and rigorously?

Reviewer #1: Yes

Reviewer #2: Yes

Reviewer #3: I Don't Know

4. Have the authors made all data underlying the findings in their manuscript fully available?

The PLOS Data policy requires authors to make all data underlying the findings described in their manuscript fully available without restriction, with rare exception (please refer to the Data Availability Statement in the manuscript PDF file). The data should be provided as part of the manuscript or its supporting information, or deposited to a public repository. For example, in addition to summary statistics, the data points behind means, medians and variance measures should be available. If there are restrictions on publicly sharing data—e.g. participant privacy or use of data from a third party—those must be specified.

Reviewer #1: Yes

Reviewer #2: Yes

Reviewer #3: Yes

5. Is the manuscript presented in an intelligible fashion and written in standard English?

PLOS ONE does not copyedit accepted manuscripts, so the language in submitted articles must be clear, correct, and unambiguous. Any typographical or grammatical errors should be corrected at revision, so please note any specific errors here.

Reviewer #1: Yes

Reviewer #2: (No Response)

Reviewer #3: No

6. Review Comments to the Author

Please use the space provided to explain your answers to the questions above. You may also include additional comments for the author, including concerns about dual publication, research ethics, or publication ethics. (Please upload your review as an attachment if it exceeds 20,000 characters)

Reviewer #1: (No Response)

Reviewer #2: All comments have been addressed and I believe this manuscript is a nice contribution to the field. Congratulations!

Reviewer #3: This is a thorough revision that has addressed all my concerns regarding the science aspects of the manuscript. I notice that the English of the manuscript still has very noticeable weaknesses. Hence, I would suggest having a native speaker revise the language prior to acceptance.

**Reply to the reviewers:**

Thank you for your thorough reviews of the revised manuscript and for providing encouraging comments and suggestions to improve its readability. We sought assistance from a native English speaker to proofread the manuscript. We hope that this revised version meets your expectations.
